# Diagnosis of Idiopathic Pulmonary Fibrosis “Pragmatic Challenges in Clinical Practice”

**DOI:** 10.3389/fmed.2017.00151

**Published:** 2017-09-20

**Authors:** Vasilios Tzilas, Argyris Tzouvelekis, Serafim Chrysikos, Spyridon Papiris, Demosthenes Bouros

**Affiliations:** ^1^First Academic Department of Pneumonology, Hospital for Thoracic Diseases, “Sotiria”, Medical School, National and Kapodistrian University of Athens, Athens, Greece; ^2^Division of Immunology, Biomedical Sciences Research Center “Alexander Fleming”, Athens, Greece; ^3^5th Department of Pneumonology, Hospital for Thoracic Diseases, “Sotiria”, Athens, Greece; ^4^2nd Pulmonary Medicine Department, Attikon University Hospital, Medical School, National and Kapodistrian University of Athens, Athens, Greece

**Keywords:** idiopathic pulmonary fibrosis, diagnosis, challenges, pragmatic, clinical practice

## Abstract

The past few years have signaled a major breakthrough on the management of idiopathic pulmonary fibrosis (IPF). Finally, we have drugs in our arsenal able to slow down the inexorable disease natural course. On the other hand, the latter evidence has increased the responsibility for a timely and accurate diagnosis. Establishment of IPF diagnosis directly affects the choice of appropriate treatment. The current diagnostic guidelines represent a major step forward providing an evidence-based road map; yet, clinicians are encountering major diagnostic dilemmas that inevitably affect therapeutic decisions. This review article aims to summarize the current state of knowledge on the diagnostic procedure of IPF based on the current guidelines and discuss pragmatic difficulties and challenges encountered by clinicians with regards to their applicability in the everyday clinical practice.

## Introduction

Idiopathic pulmonary fibrosis (IPF) represents the most common form of idiopathic interstitial pneumonia (IIPs) and is characterized by the gravest prognosis with a median survival of 3–5 years (1), irrespective of treatment. The past 10 years, large multicenter placebo-controlled clinical trials have significantly shifted the therapeutic dial of IPF (2–4) from harmful agents to Ref. (5) to therapies able to slow down the disease progression (2–4). It is important to note that the efficacy of the antifibrotic agents, pirfenidone, and nintedanib has been tested only in the context of IPF; thus, accurate and timely diagnosis seems to be imperative. It is important to note that disease diagnosis does not represent anymore an academic exercise since it directly influences and guides therapeutic decisions.

## Diagnosis of IPF

Histologically, IPF is characterized by the pattern of usual interstitial pneumonia (UIP), which is denoted by spatial and temporal heterogeneity (6). It is of utmost importance to highlight the fact that UIP pathology is not exclusive to IPF, as it may characterize other diseases including chronic hypersensitivity pneumonitis (HP), connective tissue disorders-associated ILDs, asbestosis, or drug toxicity. In other words, all IPF have UIP pathology, but not all UIP are IPF. High-resolution computed tomography (HRCT) revolutionized the diagnosis of IPF. The presence of honeycombing in a predominantly peripheral and bibasilar distribution has a sufficient positive-predictive value (PPV) for underlying UIP pathology obviating the need for tissue confirmation (7–9). Even in that case, exclusion of other causes of UIP is mandatory to finally establish IPF diagnosis. Furthermore, IPF diagnosis represents a dynamic process and, therefore, close monitoring of the patient is mandatory. In particular, therapeutic decisions can be altered based on disease natural course and treatment responsiveness on an individual basis. In addition, disease diagnosis could be revisited in light of emerging symptoms compatible with connective tissue disorder or exposure to potentially harmful environmental agents.

The patient with IPF typically presents with progressive dyspnea on exertion of insidious onset and non-productive cough. The most characteristic clinical finding is the presence of Velcro rales that can be proven an extremely useful diagnostic tool for early disease diagnosis (10). Clubbing is almost found in 30–50% of patients; yet, its prevalence is much higher following disease progression and development of pulmonary hypertension. Multisystemic manifestations in the context of IPF are highly uncommon and should alert the physician toward alternative diagnoses. Median time from onset of symptoms to first evaluation in an ILD center quite often exceeds 1 year and the length of delay has been associated with increased mortality (11, 12).

Pulmonary function tests usually exhibit a restrictive pattern with concomitant reduction in diffusing capacity of carbon monoxide (DLco). However, the absence of restriction does not exclude the diagnosis of IPF, especially in the context of combined pulmonary fibrosis and emphysema, which is characterized by relatively preserved lung volumes with disproportionately reduced DLco (13). There are also cases discovered early and presumably with an initial FVC > 100% that do not fulfill the criteria of a restrictive pattern, nevertheless, fibrosis is evident on HRCT.

According to current diagnostic criteria (1), HRCT plays a pivotal role in the diagnostic procedure. There are three diagnostic categories based on HRCT appearance: UIP pattern, possible UIP pattern, and inconsistent with UIP. The definition of each category is based on morphological as well as distribution characteristics. UIP pattern is characterized by the presence of reticular abnormalities and honeycombing (with or without traction bronchiectasis) with a subpleural and basal predominance in the absence of inconsistent features. The above radiological features in the absence of honeycombing constitute the possible UIP pattern. Inconsistent features can be categorized as those involving distribution (upper or mid lung and peribronchovascular predominance) and those involving morphology (extensive ground glass opacities, consolidation, mosaic attenuation, nodules, discrete cysts).

The presence of honeycombing is not synonymous with IPF, as it can be seen in other diseases as chronic HP, collagen tissue-related interstitial lung diseases, asbestosis, drug-induced lung toxicity, sarcoidosis, postradiation pneumonitis, and post ARDS. Minimal honeycombing can also be encountered in cases of fibrotic NSIP, which represents a major component of the differential list.

The distribution of honeycombing can offer significant information. Typically, in IPF, it has a subpleural and basilar distribution. Chronic HP can be a great mimic of IPF. However, sometimes in chronic HP, honeycombing can be more marked in the upper/mid lung zones giving a hint to the actual diagnosis. The same upper lobe predominance of honeycombing can be seen in sarcoidosis as well. Furthermore, in sarcoidosis, the fibrotic process often follows the expected perilymphatic route, thus creating a “swath” of honeycombing extending from the hilum to the periphery of the lung. In patients who develop fibrosis post ARDS, it has a striking anterior distribution (Table 1).

**Table 1:** Differential diagnosis of radiological honeycombing.

Causes of honeycombing	Comments
Idiopathic pulmonary fibrosis	Distribution predominantly bibasilar and subpleural
Collagen tissue disease	Honeycombing mainly seen in rheumatoid arthritis-ILD
Chronic hypersensitivity pneumonitis	Honeycombing can be seen predominantly in the upper/middle zones. Clues to diagnosis: mosaic attenuation, air trapping in expiratory scans
Asbestosis	Distribution predominantly bibasilar and subpleural. Clues to diagnosis: pleural plaques (±calcification), subpleural lines
NSIP	When seen, honeycombing is minimal. Clues to diagnosis: subpleural sparing, central/peribronchovascular predominance of findings
Sarcoidosis	In rare cases, the distribution is bibasilar and subpleural. Typically, honeycombing is seen in upper/middle zones extending from the periphery toward the hilum. Clues to diagnosis: perilymphatic nodules, hilar/mediastinal lymphadenopathy (±calcification), progressive massive fibrosis
Radiation	Confined to radiation port
End-stage ARDS	Usually involves anterior lung (barotrauma)
Drug toxicity	

Besides the distribution of honeycombing, there are other findings on HRCT that increase suspicion toward certain diagnoses. Silva et al. (14) studied 66 patients with biopsy proven IPF, HP, and NSIP. The presence of lobular areas with decreased attenuation, centrilobular nodules, and cysts favored the diagnosis of HP. The best predictors of NSIP were the presence of subpleural sparing and the absence of honeycombing.

In the appropriate clinical setting in patients with a UIP pattern, IPF diagnosis can be established without the need for surgical lung biopsy (SLB). In the cases of possible UIP and inconsistent with UIP, a SLB is recommended in order to reach a final diagnosis. When SLB is necessary, close cooperation with the thoracic surgeon is necessary in order to point the optimal sites for biopsy in order to increase the possibility of an accurate diagnosis. Areas of honeycombing should be avoided in lung sampling since they may reveal non-specific end-stage lung damage and absence of spatial and temporal heterogeneity suggestive of UIP. Samples should be obtained from at least two different lobes, because of the possibility of discordant findings (UIP in one lobe and NSIP in another). In such cases, the UIP pattern drives diagnosis and prognosis as well (15, 16).

Regarding histopathology, current guidelines have classified patients into four categories: UIP, probable UIP, possible UIP, and not UIP (1). Specific combinations of HRCT and SLB pattern with the integration of clinical data are evaluated by a multidisciplinary (MDT) team in order to achieve a final diagnosis.

MDT approach is acknowledged as a major advance in IPF diagnosis. It refers to the constructive exchange of views between a respiratory physician, radiologist, and pathologist with expertise in the field of ILDs. The added value of MDT diagnosis is its association with higher levels of diagnostic confidence and better interobserver agreement (17, 18). Walsh et al. (19) were the first to evaluate the agreement between different multidisciplinary teams in diffuse lung diseases after the 2013 ATS/ERS update (20) on the classification of IIPs. Inter-MDT agreement was acceptable for cases of IPF (weighted kappa coefficient, κw = 0.71), while it was moderate for NSIP (κw = 0.42) and rather disappointing for HP (κw = 0.29) reflecting the lack of diagnostic guidelines for the last two clinical entities. This indirectly impacts the accuracy of IPF diagnosis as well, given the fact that NSIP and HP are frequently major components of its differential.

## A Pragmatic Application of Guidelines in Every Day Clinical Practice

The 2011 guidelines are a clear step forward considering that they provide clear guidance on an evidence-based approach. The most crucial caveat of these guidelines is that, in a significant percentage of “real-life” patients with IPF, lack clinical practicality regarding diagnosis, prognosis, and therapeutic decisions.

### Challenge 1: Interpretation of HRCT

High-resolution computed tomography plays a pivotal role in disease diagnosis and determines the need of SLB to establish a definite diagnosis. However, accurate identification of honeycombing is not straightforward even amongst thoracic radiologists. Interobserver agreement has been proven poor, and this has been validated in recent studies (21–24). This problem is further accentuated on a community level.

#### Possible UIP Pattern

An increasing number of studies has shown that in patients with a high suspicion of IPF, a possible UIP pattern retains sufficient PPV for underlying UIP pathology in order to obviate the need for tissue based diagnosis. Raghu et al. (25) studied 315 patients with IPF study that had both HRCT and SLB samples. As expected, UIP pattern had a high PPV for UIP pathology (97.3%). This high PPV was retained for patients with a possible UIP pattern (94%). Though a selection bias is quite obvious given that this evidence refers to patients screened for recruitment into clinical trials and thus should not be generalized; yet, this study highlights the importance of pretest clinical probability. Chung et al. (26) studied 201 patients with pulmonary fibrosis that were subjected to lung biopsy within 1 year of chest CT. Patients with possible UIP on CT scan were more likely to have histologic probable/definite UIP comparing to patients with indeterminate UIP on CT scan (82.4 vs 54.2%, *p* = 0.01). Finally, in the INPULSIS trials, a significant proportion of patients (31.9%, *n* = 338) were enrolled based on possible UIP pattern (with traction bronchiectasis) and no confirmation by SLB. This group of patients exhibited the same progression of disease based on the annual decline of FVC compared to patients with honeycombing on HRCT and/or confirmation by SLB as well as similar treatment response to nintedanib (27). This observation adds further to the notion that in the appropriate clinical setting, possible UIP pattern carries sufficient PPV for UIP pathology.

Recently, a study by Brownell et al. offered valuable new information on this topic (28). Avoiding selection bias, they elegantly showed that the PPV of possible UIP for predicting UIP pathology directly depends on the pretest probability of IPF and the prevalence of IPF in the examined population. In the derivation cohort, possible UIP had a specificity of 91.2% and a PPV of 62.5%. By using two key clinical predictors (male sex, increasing age) and a radiographic predictor (total traction bronchiectasis score), the PPV increased above the acceptable threshold of 90%.

#### Inconsistent with UIP Pattern

**Figure 1:**
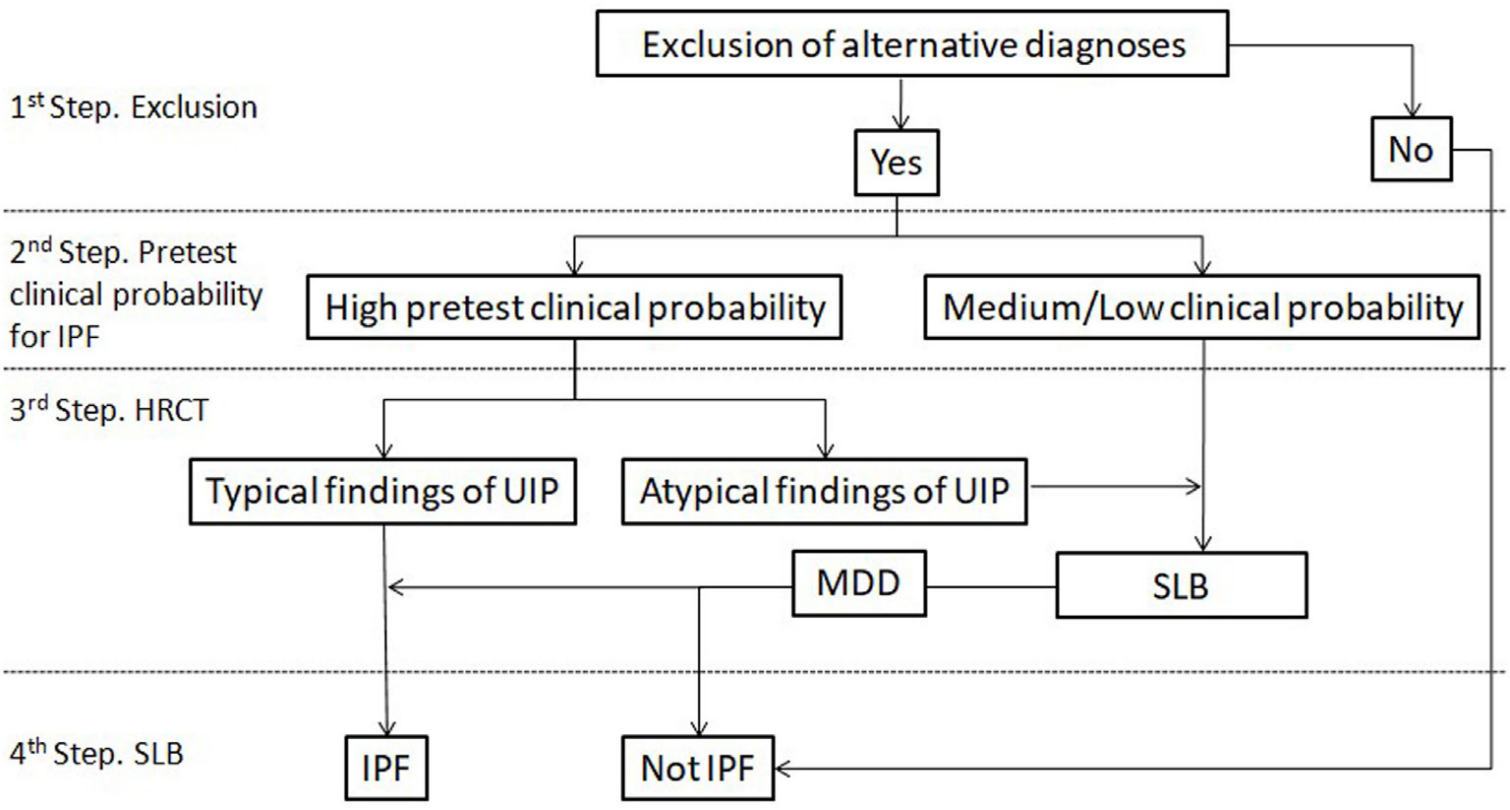
Proposed diagnostic algorithm for idiopathic pulmonary fibrosis (IPF). Typical findings of UIP are radiological signs of lung fibrosis (honeycombing, traction bronchiolectasis, and/or irregular reticular pattern) with a predominantly peripheral/subpleural and bibasilar distribution. Atypical findings of UIP are according to the current definition of inconsistent with UIP pattern. HRCT, high-resolution computed tomography; SLB, surgical lung biopsy.

According to guidelines, even if histology is that of a typical UIP pattern when the HRCT appearance is inconsistent with UIP, the diagnosis of IPF is deemed only to be possible. However, the term inconsistent seems to be a misnomer. IPF is actually a great mimic. UIP pathology can exhibit a wide variety of radiological expressions ranging from the typical UIP pattern with peripheral, bibasilar honeycombing, to a pattern resembling HP with areas of mosaic attenuation or to a pattern characterized by extensive ground glass opacities (29, 30). Interestingly, NSIP pathology seems to be much more consistent regarding its radiological expression (31). The key point is that an inconsistent with UIP radiological pattern does not rule out the diagnosis of IPF, but mandates histological confirmation regardless the pretest probability of the patient. In the study by Brownell et al., the maximum PPV achieved for the inconsistent UIP pattern regarding IPF diagnosis was just 38% (28). Figure 1 summarizes a proposed algorithm for IPF diagnosis based on recent findings as described previously.

### Challenge 2: Interpretation of SLB

The interpretation of histological findings is subject to the same limitations as HRCT. The pathologic distinction between UIP and fibrotic NSIP can be especially difficult and is the main reason (>50%) for interobserver variation in the evaluation of diffuse parenchymal lung disease (32). In the same study, the median prevalence for diagnoses with low confidence (<70% likelihood) was 18% while diagnoses made with 100% confidence were reached in only 39% of cases. Thus, it is not surprising that the pathological diagnosis can be reconsidered in almost 20% of cases following integration of clinical and HRCT data (17). The variability in interpretation is accentuated between thoracic and general pathologists with a poor level of agreement (κ: 0.21), which was shown to have direct clinical implications (33). Also, it is known that biopsies from different sites can produce discordant results, specifically, NSIP vs UIP histology (15, 16). Consequently, sampling error is a possibility and in cases with a definite UIP radiology pattern and NSIP on histology, the radiologic diagnosis actually prevails over histology (34). Finally, histology patterns in interstitial lung diseases are not exclusive to certain diseases. In fact, the same histology can correspond to different diseases and furthermore to the same disease but with strikingly different progression and natural course. SLB is not a gold standard and the risk vs benefit ratio should be carefully examined in each patient.

### Challenge 3: Safety of SLB

Surgical lung biopsy in patients with ILDs as it can trigger an acute exacerbation (35, 36), regardless disease severity (37). An alarming observation is that the same parameters that increase the likelihood of IPF (increased age and male sex) (28, 38, 39) represent risk factors that increase mortality following SLB in patients with ILDs (40, 41). Actually, a provisional diagnosis of IPF was identified as a risk factor for increased mortality. Other risk factors are the presence of comorbidities, hypoxemic respiratory failure, severe physiological impairment, pulmonary hypertension, rapidly progressive disease (42).

Two large series (40, 41) reported postoperative hospital mortality rates (1.7%) similar to those reported in patients with lung cancer undergoing lobectomy. The actual postoperative mortality may vary depending on the nature of the procedure (elective vs non-elective) and the risk factors for the individual patient. Thus, the decision to proceed to SLB [*via* video-assisted thoracoscopic surgery (VATS)] should be carefully considered on an individual basis, weighing risks vs diagnostic benefit.

### Challenge 4: Current Clinical Practice

By strictly adhering to current guidelines, a large number of patients (almost 50% with a suspicion of IPF) will need to be subjected to SLB. Clinical practice seems to have endorsed the facts that SLB carries a small but significant risk and that the possible UIP pattern in a patient with a high pretest probability of IPF retains sufficient PPV for UIP pathology. It is common practice that in patients with a high clinical probability of IPF (28, 38, 39) (male sex, increased age, and/or extent of fibrosis), we establish a working diagnosis of IPF (43, 44) without resorting to surgical biopsy. Biopsies are reserved for patients in whom establishing a tissue-based diagnosis is clinically meaningful and are fit enough to undergo such a procedure. This is vividly depicted in the study by Hutchinson et al. (40). In a UK study held between 1997 and 2008, only 4.5% of new cases with a provisional diagnosis J84.1 were subjected to SLB (40). Given the fact that the ICD-10 of J84.1 does not accurately describe the IPF population (45, 46), this percentage is likely to be even smaller for actual IPF cases. We eagerly wait to see how the above will be translated in the upcoming guidelines for the diagnosis of IPF.

## Future Directions

### Bronchoscopic Lung Cryobiopsy (BLC)

Bronchoscopic lung cryobiopsy is dynamically emerging during the past few years as an alternative diagnostic tool to SLB, claiming the same diagnostic efficacy and reduced mortality (47). Cryobiopsies are considerably larger and have minimal crash artifacts as opposed to forceps biopsies when performed by experienced bronchoscopists in appropriate organized centers. Therefore, they allow confident recognition of histological patterns. The vital question that should be addressed is their safety and diagnostic yield against lung biopsies obtained *via* VATS. A recent meta-analysis including 16 studies with BLC (642 patients) and 14 studies with VATS (1,594 patients) (48) reported comparable diagnostic yields for BLC (83.7%) and VATS (92.7%). With regards to safety profile, BLC was associated with severe bleeding in 4.9% of cases and pneumothorax in 9.5% of cases while short-term mortality was similar between BLC (0.7%) and VATS (1.8%). Similar findings have been demonstrated by earlier studies (49, 50). In order to generalize the use of BLC beyond expert centers, it is important to standardize the procedure (e.g., size of cryoprobe, number and site of biopsies, degree of sedation), offer proper training, since it is an operator-dependent procedure (51) and prospectively evaluate safety and diagnostic profile of BLC as opposed to VATS.

### Biomarkers

According to the Bayesian diagnostic approach of IPF, it would be very useful to have biomarkers that would increase the pretest probability of IPF. Ideally, these biomarkers would not only have diagnostic value but would also offer clinically relevant prognostic information regarding not only the natural course of the disease but also response to therapy. While for pulmonary embolism, d-dimers are an established diagnostic indicator in a complex disease as IPF, it is unlikely that just one “diagnosticator” will suffice. Considering that IPF diagnosis represent the least critical question for clinicians (given the major improvements of HRCT), in the real-world clinical practice, an ideal biomarker would be the one who could fulfill the unmet need for timely prediction of disease progressiveness and treatment responsiveness. In line with this, most of the studied biomarkers were mostly used as disease prognosticators rather than disease-specific diagnostic tools. Matrix metalloproteinase-(MMP)-7 represents the most extensively studied molecular biomarker that showed promising prognostic value in several independent cohorts of patients with IPF. Despite the fact that elevated MMP-7 levels clearly discriminated patients with IPF from those with HP (52); yet, they showed lack of discriminatory ability between IPF and RA-ILD (53). Further studies using highly standardized sample collection procedures and collection matrices are needed to produce reproducible and reliable diagnostic and prognostic cutoff thresholds (54). The latter observation represents an amenable need for precision medicine approaches (55, 56).

### Conclusion

The ILD community has made significant progress in understanding IPF. With the development of antifibrotic agents, accurate diagnosis is crucial. Guidance is needed to focus on practical implementation of current guidelines in a real-world clinical setting. An integral first step of the diagnostic process is the exclusion of alternative diagnoses. Equally important is the definition of the pretest probability for every patient with suspected IPF. The diagnostic significance of possible or even definite UIP pattern is completely different when facing a 45-year-old female or a 70-year-old male. Possible UIP pattern seems to carry sufficient PPV in patients with a high pretest probability of IPF. SLB should be considered in patients with inconsistent with UIP pattern after evaluating the individualized benefit risk ratio (Table 2). BLC seems an attractive, safer alternative to SLB; yet, standardization and prospective evaluation of the process is required in order to “escape” from expert centers and be embraced by common practice. Finally, biomarkers are sorely needed to fulfill the unmet need of current clinical practice: early prediction of disease progressiveness and treatment responsiveness that will timely guide therapeutic decisions.

**Table 2:** Key points for idiopathic pulmonary fibrosis diagnosis.

Exclusion of other causes is mandatory Equally important is to define pretest clinical probability based on age, sex, extend of fibrosis Even in the absence of honeycombing, in a patient with a high pretest clinical probability, the presence of other signs of lung fibrosis (traction bronchiolectasis, irregular reticulation) in a predominantly bibasilar/subpleural distribution have sufficient positive-predictive value for usual interstitial pneumonia (UIP) pathology Atypical features (inconsistent with UIP) mandates the need for surgical lung biopsy (SLB), regardless pretest clinical probability SLB is not a diagnostic panacea, it can be potentially hazardous especially when performed non-electively and should be performed after carefully estimating the clinically meaningful benefit vs risk on an individual basis

## Author Contributions

VT, AT, and SC wrote the manuscript. SP and DB revised the manuscript for important intellectual content.

## Conflict of Interest Statement

The authors declare that the research was conducted in the absence of any commercial or financial relationships that could be construed as a potential conflict of interest.

## References

[1] Raghu G Collard HR Egan JJ Martinez FJ Behr J Brown KK An official ATS/ERS/JRS/ALAT statement: idiopathic pulmonary fibrosis: evidence-based guidelines for diagnosis and management. Am J Respir Crit Care Med (2011) 183:788–824. 10.1164/rccm.2009-040GL 21471066 PMC5450933

[2] Noble PW Albera C Bradford WZ Pirfenidone in patients with idiopathic pulmonary fibrosis (CAPACITY): two randomized trials. Lancet (2011) 377:1760–9. 10.1016/S0140-6736(11)60405-4 21571362

[3] King TE Jr Bradford WZ Castro-Bernardini S Fagan EA Glaspole I Glassberg MK A phase 3 trial of pirfenidone in patients with idiopathic pulmonary fibrosis. N Engl J Med (2014) 370:2083–92. 10.1056/NEJMoa1402582 24836312

[4] Richeldi L du Bois RM Raghu G Azuma A Brown KK Costabel U Efficacy and safety of nintedanib in idiopathic pulmonary fibrosis. N Engl J Med (2014) 370:2071–82. 10.1056/NEJMoa1402584 24836310

[5] Idiopathic Pulmonary Fibrosis Clinical Research Network Raghu G Anstrom KJ King TE Jr Lasky JA Martinez FJ. Prednisone, azathioprine, and N-acetylcysteine for pulmonary fibrosis. N Engl J Med (2012) 366(21):1968–77. 10.1056/NEJMoa1113354 22607134 PMC3422642

[6] Smith M Dalurzo M Panse P Parish J Leslie K. Usual interstitial pneumonia-pattern fibrosis in surgical lung biopsies. Clinical, radiological and histopathological clues to aetiology. J Clin Pathol (2013) 66(10):896–903. 10.1136/jclinpath-2013-201442 23703852 PMC3786616

[7] Raghu G Mageto YN Lockhart D Schmidt RA Wood DE Godwin JD. The accuracy of the clinical diagnosis of new-onset idiopathic pulmonary fibrosis and other interstitial lung disease: a prospective study. Chest (1999) 116:1168–74. 10.1378/chest.116.5.1168 10559072

[8] Hunninghake GW Zimmerman MB Schwartz DA King TE Jr Lynch J Hegele R Utility of a lung biopsy for the diagnosis of idiopathic pulmonary fibrosis. Am J Respir Crit Care Med (2001) 164:193–6. 10.1164/ajrccm.164.2.2101090 11463586

[9] Flaherty KR Thwaite EL Kazerooni EA Gross BH Toews GB Colby TV Radiological versus histological diagnosis in UIP and NSIP: survival implications. Thorax (2003) 58:143–8. 10.1136/thorax.58.2.143 12554898 PMC1746568

[10] Cottin V Cordier JF Velcro crackles: the key for early diagnosis of idiopathic pulmonary fibrosis? Eur Respir J (2012) 40(3):519–21. 10.1183/09031936.00001612 22941541

[11] Purokivi M Hodgson U Myllärniemi M Salomaa ER Kaarteenaho R. Are physicians in primary health care able to recognize pulmonary fibrosis? Eur Clin Respir J (2017) 4(1):1290339. 10.1080/20018525.2017.1290339 28326180 PMC5345592

[12] Lamas DJ Kawut SM Bagiella E Philip N Arcasoy SM Lederer DJ. Delayed access and survival in idiopathic pulmonary fibrosis: a cohort study. Am J Respir Crit Care Med (2011) 184(7):842–7. 10.1164/rccm.201104-0668OC 21719755 PMC3208648

[13] Cottin V Nunes H Brillet PY Delaval P Devouassoux G Tillie-Leblond I Combined pulmonary fibrosis and emphysema: a distinct underrecognised entity. Eur Respir J (2005) 26:586–93. 10.1183/09031936.05.00021005 16204587

[14] Silva CI Müller NL Lynch DA Curran-Everett D Brown KK Lee KS Chronic hypersensitivity pneumonitis: differentiation from idiopathic pulmonary fibrosis and nonspecific interstitial pneumonia by using thin-section CT. Radiology (2008) 246(1):288–97. 10.1148/radiol.2453061881 18096541

[15] Flaherty KR Travis WD Colby TV Toews GB Kazerooni EA Gross BH Histopathologic variability in usual and nonspecific interstitial pneumonias. Am J Respir Crit Care Med (2001) 164:1722–7. 10.1164/ajrccm.164.9.2103074 11719316

[16] Monaghan H Wells AU Colby TV du Bois RM Hansell DM Nicholson AG. Prognostic implications of histologic patterns in multiple surgical lung biopsies from patients with idiopathic interstitial pneumonias. Chest (2004) 125:522–6. 10.1378/chest.125.2.522 14769733

[17] Flaherty KR King TE Jr Raghu G Lynch JP 3rd Colby TV Travis WD Idiopathic interstitial pneumonia: what is the effect of a multidisciplinary approach to diagnosis? Am J Respir Crit Care Med (2004) 170:904–10. 10.1164/rccm.200402-147OC 15256390

[18] Thomeer M Demedts M Behr J Buhl R Costabel U Flower CD Multidisciplinary interobserver agreement in the diagnosis of idiopathic pulmonary fibrosis. Eur Respir J (2008) 31:585–91. 10.1183/09031936.00063706 18057059

[19] Walsh SL Wells AU Desai SR Poletti V Piciucchi S Dubini A Multicentre evaluation of multidisciplinary team meeting agreement on diagnosis in diffuse parenchymal lung disease: a case-cohort study. Lancet Respir Med (2016) 4(7):557–65. 10.1016/S2213-2600(16)30033-9 27180021

[20] Travis WD Costabel U Hansell DM King TE Jr Lynch DA Nicholson AG An official American Thoracic Society/European Respiratory Society statement: update of the international multidisciplinary classification of the idiopathic interstitial pneumonias. Am J Respir Crit Care Med (2013) 188(6):733–48. 10.1164/rccm.201308-1483ST 24032382 PMC5803655

[21] Lynch DA Godwin JD Safrin S Starko KM Hormel P Brown KK Idiopathic Pulmonary Fibrosis Study Group. High-resolution computed tomography in idiopathic pulmonary fibrosis: diagnosis and prognosis. Am J Respir Crit Care Med (2005) 17:488–93. 10.1164/rccm.200412-1756OC 15894598

[22] Watadani T Sakai F Johkoh T Noma S Akira M Fujimoto K Interobserver variability in the CT assessment of honeycombing in the lungs. Radiology (2013) 26:936–44. 10.1148/radiol.12112516 23220902

[23] Sundaram B Gross BH Martinez FJ Oh E Müller NL Schipper M Accuracy of high-resolution CT in the diagnosis of diffuse lung disease: effect of predominance and distribution of findings. AJR Am J Roentgenol (2008) 191(4):1032–9. 10.2214/AJR.07.3177 18806139

[24] Walsh SL Calandriello L Sverzellati N Wells AU Hansell DM UIP Observer Consort. Interobserver agreement for the ATS/ERS/JRS/ALAT criteria for a UIP pattern on CT. Thorax (2016) 7:45–51. 10.1136/thoraxjnl-2015-207252 26585524

[25] Raghu G Lynch D Godwin JD Webb R Colby TV Leslie KO Diagnosis of idiopathic pulmonary fibrosis with high-resolution CT in patients with little or no radiological evidence of honeycombing: secondary analysis of a randomised, controlled trial. Lancet Respir Med (2014) 2:277–84. 10.1016/S2213-2600(14)70011-6 24717624

[26] Chung JH Chawla A Peljto AL Cool CD Groshong SD Talbert JL CT scan findings of probable usual interstitial pneumonitis have a high predictive value for histologic usual interstitial pneumonitis. Chest (2015) 147:450–9. 10.1378/chest.14-0976 25317858 PMC4314819

[27] Raghu G Wells AU Nicholson AG Richeldi L Flaherty KR Le Maulf F Effect of nintedanib in subgroups of idiopathic pulmonary fibrosis by diagnostic criteria. Am J Respir Crit Care Med (2017) 195(1):78–85. 10.1164/rccm.201602-0402OC 27331880 PMC5214917

[28] Brownell R Moua T Henry TS Elicker BM White D Vittinghoff E The use of pretest probability increases the value of high-resolution CT in diagnosing usual interstitial pneumonia. Thorax (2017) 72(5):424–9. 10.1136/thoraxjnl-2016-209671 28082530 PMC5555580

[29] Sverzellati N Wells AU Tomassetti S Desai SR Copley SJ Aziz ZA Biopsy-proved idiopathic pulmonary fibrosis: spectrum of nondiagnostic thin-section CT diagnoses. Radiology (2010) 254(3):957–64. 10.1148/radiol.0990898 20177106

[30] Yagihashi K Huckleberry J Colby TV Tazelaar HD Zach J Sundaram B Radiologic-pathologic discordance in biopsy-proven usual interstitial pneumonia. Eur Respir J (2016) 47(4):1189–97. 10.1183/13993003.01680-2015 26917616

[31] Sumikawa H Johkoh T Fujimoto K Arakawa H Colby TV Fukuoka J Pathologically proved nonspecific interstitial pneumonia: CT pattern analysis as compared with usual interstitial pneumonia CT pattern. Radiology (2014) 272(2):549–56. 10.1148/radiol.14130853 24661246

[32] Nicholson AG Addis BJ Bharucha H Clelland CA Corrin B Gibbs AR Interobserver variation between pathologists in diffuse parenchymal lung disease. Thorax (2004) 59:500–5. 10.1136/thx.2003.011734 15170033 PMC1747021

[33] Lettieri CJ Veerappan GR Parker JM Franks TJ Hayden D Travis WD Discordance between general and pulmonary pathologists in the diagnosis of interstitial lung disease. Respir Med (2005) 99(11):1425–30. 10.1016/j.rmed.2005.03.008 16210097

[34] Zander DS Idiopathic interstitial pneumonias and the concept of the trump card. Chest (2004) 125(2):359–60. 10.1378/chest.125.2.359 14769707

[35] Utz JP Ryu JH Douglas WW Hartman TE Tazelaar HD Myers JL High short-term mortality following lung biopsy for usual interstitial pneumonia. Eur Respir J (2001) 17:175–9. 10.1183/09031936.01.17201750 11334116

[36] Kreider ME Hansen-Flaschen J Ahmad NN Rossman MD Kaiser LR Kucharczuk JC Complications of video-assisted thoracoscopic lung biopsy in patients with interstitial lung disease. Ann Thorac Surg (2007) 83:1140–4. 10.1016/j.athoracsur.2006.10.002 17307476

[37] Chida M Ono S Hoshikawa Y Kondo T. Subclinical idiopathic pulmonary fibrosis is also a risk factor of postoperative acute respiratory distress syndrome following thoracic surgery. Eur J Cardiothorac Surg (2008) 34:878–81. 10.1016/j.ejcts.2008.07.028 18722134

[38] Fell CD Martinez FJ Liu LX Murray S Han MK Kazerooni EA Clinical predictors of a diagnosis of idiopathic pulmonary fibrosis. Am J Respir Crit Care Med (2010) 181(8):832–7. 10.1164/rccm.200906-0959OC 20056903 PMC2854332

[39] Salisbury ML Xia M Murray S Bartholmai BJ Kazerooni EA Meldrum CA Predictors of idiopathic pulmonary fibrosis in absence of radiologic honeycombing: a cross sectional analysis in ILD patients undergoing lung tissue sampling. Respir Med (2016) 118:88–95. 10.1016/j.rmed.2016.07.016 27578476 PMC5008035

[40] Hutchinson JP McKeever TM Fogarty AW Navaratnam V Hubbard RB. Surgical lung biopsy for the diagnosis of interstitial lung disease in England: 1997-2008. Eur Respir J (2016) 48(5):1453–61. 10.1183/13993003.00378-2016 27660509

[41] Hutchinson JP Fogarty AW McKeever TM Hubbard RB. In-hospital mortality after surgical lung biopsy for interstitial lung disease in the United States 2000 to 2011. Am J Respir Crit Care Med (2016) 193:1161–7. 10.1164/rccm.201508-1632OC 26646481

[42] Raj R Raparia K Lynch DA Brown KK Surgical lung biopsy for interstitial lung diseases. Chest (2017) 151(5):1131–40. 10.1016/j.chest.2016.06.019 27471113

[43] Wells AU Any fool can make a rule and any fool will mind it. BMC Med (2016) 14:23 10.1186/s12916-016-0562-1 26860705 PMC4748459

[44] Tzilas V Bouros D Usual interstitial pneumonia pattern in the diagnosis of idiopathic pulmonary fibrosis? Lancet Respir Med (2016) 4(10):770–2. 10.1016/S2213-2600(16)30231-4 27707457

[45] Tzilas V Bouros D Inherent weaknesses of the current ICD coding system regarding idiopathic pulmonary fibrosis. Eur Respir J (2015) 45(4):1194–6. 10.1183/09031936.00205914 25829436

[46] Kaunisto J Kelloniemi K Sutinen E Hodgson U Piilonen A Kaarteenaho R Re-evaluation of diagnostic parameters is crucial for obtaining accurate data on idiopathic pulmonary fibrosis. BMC Pulm Med (2015) 15:92. 10.1186/s12890-015-0074-3 26285574 PMC4541726

[47] Colby TV Tomassetti S Cavazza A Dubini A Poletti V Transbronchial cryobiopsy in diffuse lung disease: update for the pathologist. Arch Pathol Lab Med (2017) 141(7):891–900. 10.5858/arpa.2016-0233-RA 27588334

[48] Iftikhar IH Alghothani L Sardi A Berkowitz D Musani AI. Transbronchial lung cryobiopsy and video-assisted thoracoscopic lung biopsy in the diagnosis of diffuse parenchymal lung disease: a meta-analysis of diagnostic test accuracy. Ann Am Thorac Soc (2017) 14(7):1197–211. 10.1513/AnnalsATS.201701-086SR 28399377

[49] Ravaglia C Bonifazi M Wells AU Tomassetti S Gurioli C Piciucchi S Safety and diagnostic yield of transbronchial lung cryobiopsy in diffuse parenchymal lung diseases: a comparative study versus video-assisted thoracoscopic lung biopsy and a systematic review of the literature. Respiration (2016) 91(3):215–27. 10.1159/000444089 26926876

[50] Johannson KA Marcoux VS Ronksley PE Ryerson CJ. Diagnostic yield and complications of transbronchial lung cryobiopsy for interstitial lung disease. A systematic review and metaanalysis. Ann Am Thorac Soc (2016) 13:1828–38. 10.1513/AnnalsATS.201606-461SR 27466899

[51] Poletti V Hetzel J Transbronchial cryobiopsy in diffuse parenchymal lung disease: need for procedural standardization. Respiration (2015) 90(4):275–8. 10.1159/000439313 26384323

[52] Rosas IO Richards TJ Konishi K Zhang Y Gibson K Lokshin AE MMP1 and MMP7 as potential peripheral blood biomarkers in idiopathic pulmonary fibrosis. PLoS Med (2008) 5(4):e93 10.1371/journal.pmed.0050093 18447576 PMC2346504

[53] White ES Xia M Murray S Dyal R Flaherty CM Flaherty KR Plasma surfactant protein-D, matrix metalloproteinase-7, and osteopontin index distinguishes idiopathic pulmonary fibrosis from other idiopathic interstitial pneumonias. Am J Respir Crit Care Med (2016) 194(10):1242–51. 10.1164/rccm.201505-0862OC 27149370 PMC5114439

[54] Tzouvelekis A Herazo-Maya JD Slade M Chu JH Deiuliis G Ryu C Validation of the prognostic value of MMP-7 in idiopathic pulmonary fibrosis. Respirology (2017) 22(3):486–93. 10.1111/resp.12920 27761978 PMC5352520

[55] Tzouvelekis A Herazo-Maya J Sakamoto K Bouros D. Biomarkers in the evaluation and management of idiopathic pulmonary fibrosis. Curr Top Med Chem (2016) 16(14):1587–98. 10.2174/1568026616666150930120959 26420365

[56] Tzouvelekis A Tzilas V Papiris S Aidinis V Bouros D Diagnostic and prognostic challenges in idiopathic pulmonary fibrosis: a patient’s “Q and A” approach. Pulm Pharmacol Ther (2017) 42:21–4. 10.1016/j.pupt.2016.12.002 27979760

